# Adherence to guidelines for incidental pulmonary nodules: insights from a Nordic survey

**DOI:** 10.2340/1651-226X.2025.42461

**Published:** 2025-01-08

**Authors:** Kirill Neumann, Janna Berg, Haseem Ashraf, Johan Isaksson, Aija Knuuttila, Morten H. Borg, Torben R Rasmussen

**Affiliations:** a Pulmonary Department, Akershus University Hospital, Nordbyhagen, Norway; b Pulmonary Department, Vestfold Hospital Trust, Tønsberg, Norway; c Department of Diagnostic Imaging, Akershus University Hospital, Lørenskog, Norway; d Division of Medicine and Laboratory Sciences, University of Oslo, Oslo, Norway; e Centre for Research and Development, Region Gävleborg, Uppsala University, Uppsala, Sweden; f Heart- and Lung Center and Cancer Center, Helsinki University Hospital and University of Helsinki, Helsinki, Finland; g Department of Medicine, Lillebaelt Hospital Vejle, Vejle, Denmark; h Department of Respiratory Diseases and Allergy, Aarhus University Hospital, Aarhus, Denmark

**Keywords:** Pulmonary nodule, adherence to guidelines, adherence, healthcare survey, Nordic

## Abstract

**Background and purpose:**

There is limited data on the real-world management of incidental pulmonary nodules (IPN). In this article, we review current practices and adherence to international guidelines in the Nordic countries.

**Materials and methods:**

This non-interventional, observational survey study based on an online survey consisting of 13 questions. In total, 32 hospitals responded to the survey, with 11 from Denmark, 10 from Sweden, 7 from Norway, and 4 from Finland, resulting in an overall response rate of 86% (32/37). These institutions reported following a median of 20 new lung nodules monthly (5–400 IPN cases per month).

**Results:**

In Denmark and Sweden, 100% of respondents indicated the presence of national guidelines. In Norway, this rate was 86%, and in Finland 80%. Among the primary guidelines followed, 70% of respondents reported using national guidelines, 20% used international guidelines, and only 10% reported relying on local/institutional guidelines as their first choice. Most sites used a combination of international and national guidelines (75%, 24/32). Available international guidelines were equally represented, with 35% using the Fleischner Criteria, 30% using British Thoracic Society guidelines, and 35% using others (e.g. European Society for Medical Oncology, National Comprehensive Cancer Network). There was variation in which department held primary responsibility for IPN follow-up. The article also demonstrated differences in suggested follow-up cases from the survey.

**Interpretation:**

The study reveals strong adherence to guidelines among Nordic hospitals, with a notable preference for hybrid approaches that combine different guidelines. We need continued efforts to harmonize and update guidelines.

## Introduction

Incidental pulmonary nodules (IPNs) are nodules discovered unintentionally on computed tomography (CT) scans conducted for unrelated reasons such as trauma, pulmonary disease, and cardiac or mediastinal conditions. According to the Fleischner Society, an IPN refers to lung space-occupying lesions larger than 3 mm and up to 3 cm in diameter, without specific classification regarding their nature. These nodules are surrounded by lung tissue and can be solitary or multiple. IPNs are also categorized into solid and subsolid nodules, with the subsolid nodules further classified into pure ground-glass nodules and semisolid nodules.

Although the proportion of patients with IPNs varies between 10 and 59% in the literature [1, 2], it is evident that IPNs are very common in the population. Extrapolation of the data suggests that in 2015, ~1.6 million IPNs were detected by CT scans in 1 year in the entire US population [3]. CT has been increasingly used worldwide, and incidental findings are growing likewise [2, 4].

While only a small proportion of IPNs correspond to early-stage lung cancer, identifying and selecting the malignant ones is crucial. Accurate assessment and appropriate follow-up of IPNs may be just as important as screening programs in reducing lung cancer mortality. For example, in a previous study by Vindum et al., out of 4,181 patients with IPN monitored with CT scans, 249 (6%) were diagnosed with lung cancer. Of these, 224 (90%) were diagnosed during follow-up, with the majority of cases detected at an early, potentially curable stage [5]. At the same time, we know that almost half of the patients with IPNs are not appropriately monitored, and it was estimated that 2.5% of stage IV lung cancer could have been detected earlier with proper surveillance [6–8].

Despite existing guidelines from major medical societies like The Fleischner Society [9], BTS (British Thoracic Society) [10], ACCP (the American College of Chest Physicians) [11], and the NCCN (National Comprehensive Cancer Network) [12], studies suggest that many health care practitioners are not fully aware of or do not consistently apply them. Awareness and use of the guidelines vary significantly between countries, ranging from 27 to 61% in different studies [13]. The varying adherence to these guidelines among radiologists, pulmonologists, and general practitioners indicates a need for improvement.

There is limited data on the real-world management of IPNs, and clinical routines remain uncertain, leading to many potentially overlooked cases. In this article, we review current practices and adherence to international guidelines in the Nordic countries, evaluate discrepancies and knowledge gaps across these countries and provide a basis for future research and improvements in existing practices.

## Methods

This non-interventional, observational survey study aimed to evaluate current practices and procedures for IPN follow-up in the Nordic countries. The study was conducted in three phases: (1) an online workshop to define research questions and survey content, (2) a structured survey for healthcare professionals, and (3) summarization and validation of the results.

### Online workshop

A virtual, multi-professional expert workshop was conducted, involving pulmonary physicians (*n* = 5) and thoracic radiologists (*n* = 3) from Denmark and Norway, facilitated by representatives from AstraZeneca (*n* = 2). During the workshop, participants discussed existing knowledge gaps, key assessment points, and the overall coordination workflow for this project. The workshop also aimed to establish a Nordic collaboration on IPN management, leading to the future development of survey questions.

### Structured survey

Following the workshop, the survey draft was reviewed and refined based on feedback from the expert panel. The final survey consisted of 13 questions: two to identify the responding site, eight multiple-choice questions, and three open-ended questions. Five of the questions included different theoretical examples. The questionnaire is provided in the Supplementary Materials. Google Forms was used as the survey platform.

The survey was disseminated across the four most populous Nordic countries, encompassing 98.5% of the total Nordic population. The survey aimed to encompass all pulmonary departments across Denmark, Norway, Sweden, and Finland, targeting both university hospitals, central hospitals, and local hospitals in each country. The distribution was facilitated through the authors’ extensive collective network of contacts working with lung cancer and with clinical experience within the area of IPN follow-up. The selection process aimed to include various regions within each country. After an initial 3-week period, a reminder was sent to non-responding sites. Data collection spanned from early May 2024 to the end of July 2024, spanning a total of 3 months.

### Summarizing and validation

Upon receiving the survey responses, participants from the initial workshop validated the results, clarified any ambiguous data, and prioritized the most significant factors identified in the survey. Communication during this phase was conducted via email.

### Statistical approach

Numerical responses were analyzed using average values; however, statistical significance was not assessed due to the nature of the questions and the limited number of responses. Open-ended responses were analyzed qualitatively, focusing on the level of detail.

### Data availability statement

Data used in the study are available on request to corresponding author.

### Ethical considerations

All survey participants were informed about the study’s purpose, data usage, and measures to ensure privacy and confidentiality during data collection. No personal or patient-specific data were collected, thus exempting this study from the need for informed consent or patient Institutional Review Board/Ethics Committee approvals.

## Results

In total, 32 hospitals responded to the survey, with 11 from Denmark, 10 from Sweden, 7 from Norway, and 4 from Finland, resulting in an overall response rate of 86% (32/37). These institutions reported following a median of 20 new lung nodules per month, with the number ranging from 5 to 400 nodules depending on the site’s patient volume.

### IPN follow-up

There was variation in which department held primary responsibility for IPN follow-up. In 19 cases, the pulmonary department was responsible, while only one site reported that the radiology department alone handled the follow-up. The remaining respondents (*n* = 12) reported a multidisciplinary approach, with pulmonologists and radiologists making shared decisions. Primary care physicians alone were responsible in two cases; however, three additional sites reported primary care as an alternative institution for IPN follow-up, all in Sweden.

**Figure 1:**
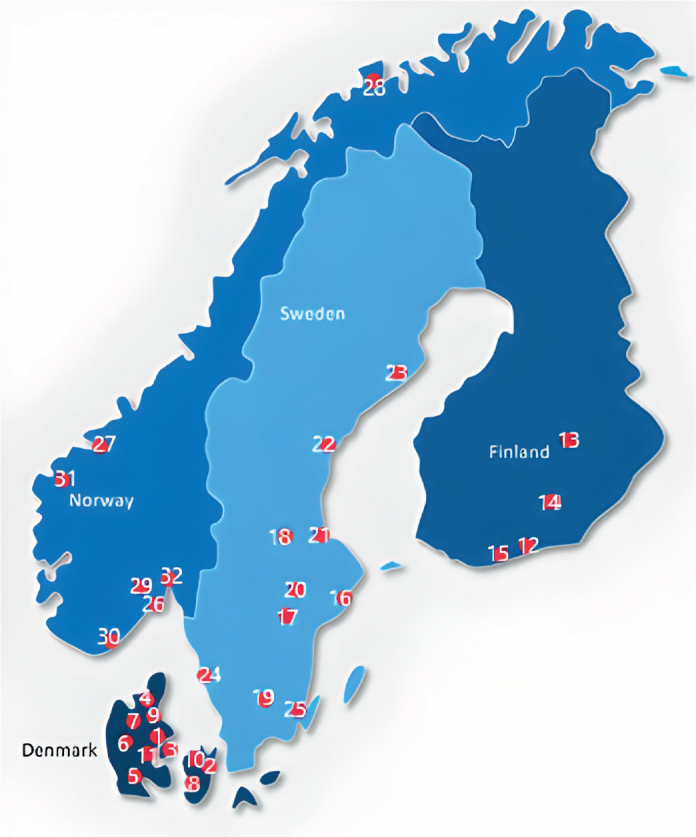
Participating hospitals (marked with red dots). **Denmark:** 1) Aarhus 2) Copenhagen 3) Odense 4) Aalborg 5) Sønderborg 6) Gødstrup 7) Viborg 8) Næstved 9) Randers 1) Roskilde 11) Vejle. **Finland**: 12) Helsinki 13) Kuopio 14) Lahti 15) Espoo. **Sweden**: 16) Stockholm 17) Eskilstuna 18) Falun 19) Växjö 20) Västerås 21) Gävle 22) Sundvall 23) Umeå 24) Gothenburg 25) Kalmar. **Norway**: 26) Tønsberg 27) Trondheim 28) Tromsø 29) Kongsberg 30) Kristiansand 31) Møre og Romsdal 32) Lørenskog

### Guidelines used

In Denmark and Sweden, 100% of respondents indicated the presence of national guidelines. In Norway, this rate was 86%, and in Finland 80%. Among the primary guidelines followed, 70% of respondents reported using national guidelines, 20% used international guidelines, and only 10% reported relying on local/institutional guidelines as their first choice. Most sites used both international and national guidelines (75%, 24/32). Available international guidelines were equally represented, with 35% using the Fleischner Criteria, 30% using BTS guidelines, and 35% using others (e.g. European Society for Medical Oncology [ESMO], NCCN).

### Management of solid lung nodules

There was some discrepancy in strategies for managing different scenarios involving solid lung nodules. For a solid lung nodule of 6 mm with no previous imaging and no typical benign morphology, most sites chose CT control at 6 months (*n* = 16), followed by 3 months (*n* = 5) or 12 months (*n* = 5). Five sites used the Brock risk score for control stratification, and one site opted for further investigation with Positron Emission Tomography (PET) and biopsy. For a solid lung nodule of 8 mm with no previous imaging and no typical benign morphology, sites chose either CT control at 6 months (*n* = 10), CT control at 3 months (*n* = 8), or further investigation with PET and biopsy (*n* = 8). An additional 6 sites would consider PET and biopsy after applying the Brock risk score. For a solid lung nodule of 10 mm without previous imaging and no benign criteria, 27 out of 32 sites chose investigation with PET and biopsy, while the remaining five sites would consider this option after applying the Brock risk score. The response option ‘Depending on Brock risk score’ in the survey was designed as a possibility to indicate a need for additional patient information, rather than relying solely on the data derived from the images, and can serve as a proxy for grade of uncertainity or complexity in this instance.

### Management of ground glass nodules

For a Ground Glass Opacity of 8 mm with no previous imaging, 60% of respondents chose CT follow-up at 3 months, while 40% opted for CT follow-up at 6 months. For a Ground Glass Opacity of 10 mm with no previous imaging, 13 respondents chose CT follow-up at 3 months, 7 chose CT follow-up at 6 months, 3 chose CT follow-up at 1 year, and 8 opted for investigation with PET and biopsy. One response was missing.

## Discussion

### Use of IPN guidelines

The survey results indicate commendable adherence to IPN guidelines among Nordic hospitals, with all responding institutions incorporating these guidelines into their everyday practice. Although established national and international recommendations for action are in place, previous studies have shown much lower rates of guideline utilization (35–60%). This discrepancy is often due to medical practitioners either being unaware of the recommendations or, despite being aware, failing to apply them correctly [14, 15]. Such lapses can lead to missed or delayed follow-ups, as well as excessive follow-ups, including unnecessary invasive procedures or nuclear imaging.

But our findings still highlight opportunities for improving IPN management. There is no standardized approach among the responding institutions, with a variety of different guidelines – sometimes used in combination – being followed. Other studies have also shown that medical experts involved in IPN follow-up express concerns about the guidelines, noting that they can be vague or not applicable to certain nodules [16]. Radiologists reported that they sometimes had to deviate from the guidelines based on their expertise in interpreting clinical history and IPN characteristics. This leads to inconsistencies in patient care, as unclear or suboptimal guidelines are thought to contribute to lower adherence. In addition, the applicability of existing guidelines can vary depending on local factors such as healthcare accessibility, socioeconomic status, and more. Given that the Nordic countries have relatively uniform healthcare systems, they might benefit from adopting a common framework rooted in existing guidelines but adapted for local conditions. The knowledge about IPNs is well-established, and current guidelines are supported by a vast database of cases. Investigating a combination of different guidelines that allow for more precise risk assessment in each case may be a reasonable approach for future studies.

### Managing solid and ground glass nodules

The management strategies for solid lung nodules and ground glass opacities varied, reflecting the complexity of clinical decision-making in these cases. For example, follow-up intervals for a 6 mm solid nodule ranged from 3 to 12 months, with some institutions opting for further investigations based on risk stratification tools like the Brock risk score. Similarly, for an 8 mm nodule, the suggested options included CT follow-up at 3 or 6 months, or PET and biopsy in certain cases, sometimes guided by Brock Risk Score. A study by Rampinelli et al. [17] showed similar variability in management strategies, particularly for smaller and ground glass lesions, where a cautious approach with shorter follow-up intervals was more common. Our study also found a more uniform approach for managing larger solid lesions.

Current IPN guidelines offer recommendations rather than strict rules, so some variation is expected in real world practice. In the current study, even in the countries with national guidelines like Denmark and Sweden, we still have differences in IPN management within that same country stressing the difficulty of interpreting the guidelines in clinical practice. These variations underscore the need for flexible yet clear guidelines that accommodate different clinical scenarios while maintaining a standardized approach, considering factors like risk factors and patient preferences. Many institutions in our survey adopted a hybrid approach, combining different international and local guidelines. This strategy allows them to incorporate global best practices while adapting to local contexts. Studies suggest that guideline application can be further improved by calculating individual malignancy risk using models like the Mayo Clinic model, the Brock model (CT) or the Herder model (CT+PET), which take into account factors such as age, sex, ethnicity, family history of lung cancer, previous extra-thoracic cancer, and/or smoking history [18, 19], but these additional information may not always be readily available.

## Strengths and limitations

This study includes hospitals with varying patient volumes, ensuring a comprehensive representation of practices across different regions in the Nordic countries. By surveying hospitals directly, it provides valuable insights into how guidelines are implemented in real-world settings. However, the study has limitations typical of self-reported data, including potential respondent bias. Additionally, hospitals that did not participate may have different practices or levels of guideline adherence, which could skew the findings. Since surveys capture practices at a single point in time, longitudinal studies would be necessary to understand how these practices evolve.

## Conclusions

The survey reveals strong adherence to guidelines among Nordic hospitals, with a notable preference for hybrid approaches that combine different guidelines. While there is room for improvement, the overall commitment to following evidence-based protocols is commendable and crucial for optimizing patient outcomes. Continued efforts to harmonize and update guidelines, along with targeted interventions to address barriers to adherence, will further enhance the quality of care for patients with incidentally detected pulmonary nodules.

## Supplementary Material

Adherence to guidelines for incidental pulmonary nodules: insights from a Nordic survey

## References

[1] Blagev D, Lloyd J, Conner K, Dickerson J, Adams D, Stevens SM, et al. Follow-up of incidental pulmonary nodules and the radiology report. J Am Coll Radiol. 2014;11(4):378–83. 10.1016/j.jacr.2013.08.003 24316231

[2] Hendrix W, Rutten M, Hendrix N, van Ginneken B, Schaefer-Prokop C, Scholten ET, et al. Trends in the incidence of pulmonary nodules in chest computed tomography: 10-year results from two Dutch hospitals. Eur Radiol. 2023;33(11):8279–88. 10.1007/s00330-023-09826-3 37338552 PMC10598118

[3] Gould M, Tang T, Liu I, Lee J, Zheng C, Danforth K, et al. Recent trends in the identification of incidental pulmonary nodules. Am J Respir Crit Care Med. 2015;192(10):1208–14. 10.1164/rccm.201505-0990OC 26214244

[4] Borg M, Hilberg O, Andersen MB, Weinreich UM, Rasmussen TR. Increased use of computed tomography in Denmark: stage shift toward early stage lung cancer through incidental findings. Acta Oncol. 2022;61(10):1256–62. 10.1080/0284186X.2022.2135134 36264585

[5] Vindum HH, Kristensen K, Christensen NL, Madsen HH, Rasmussen TR. Outcome of incidental pulmonary nodules in a real-world setting. Clin Lung Cancer. 2023;24(8):673–81. 10.1016/j.cllc.2023.09.003 37839963

[6] Lee JS, Lisker S, Vittinghoff E, Cherian R, McCoy DB, Rybkin A, et al. Follow-up of incidental pulmonary nodules and association with mortality in a safety-net cohort. Diagnosis. 2019;6(4):351–9. 10.1515/dx-2019-0008 31373897 PMC7757426

[7] Wayne MT, Prescott HC, Arenberg DA. Prevalence and consequences of non-adherence to an evidence-based approach for incidental pulmonary nodules. PLoS One. 2022;17(9):e0274107. 10.1371/journal.pone.0274107 36084105 PMC9462825

[8] Borg M, Kristensen K, Alstrup G, Mamaeva T, Arshad A, Laursen CB, et al. Consequences of losing incidental pulmonary nodules to follow-up: unmonitored nodules progressing to stage IV lung cancer. Respiration. 2024;103(2):53–9. 10.1159/000535595 38253045

[9] MacMahon H, Naidich D, Goo J, Lee K, Leung A, Mayo JR, et al. Guidelines for management of incidental pulmonary nodules detected on CT images: from the Fleischner Society 2017. Radiology. 2017;284(1):228–43. 10.1148/radiol.2017161659 28240562

[10] Callister M, Baldwin D, Akram A, Barnard S, Cane P, Draffan J, et al. The British Thoracic Society guidelines on the investigation and management of pulmonary nodules: accredited by NICE. Thorax. 2015;70(Suppl. 2):ii1–54. 10.1136/thoraxjnl-2015-207168 26082159

[11] Gould M, Donington J, Lynch W, Mazzone P, Midthun D, Naidich D, et al. Evaluation of individuals with pulmonary nodules: when is it lung cancer? Diagnosis and management of lung cancer, 3rd ed: American College of Chest Physicians evidence-based clinical practice guidelines. Chest. 2013;143(5 Suppl):e93S–120S. 10.1378/chest.12-2351 23649456 PMC3749714

[12] Wood DE, Kazerooni EA, Baum SL, Eapen GA, Ettinger DS, Hou L, et al. Lung cancer screening, version 3.2018, NCCN clinical practice guidelines in oncology. J Natl Compr Cancer Netw. 2018;16(4):412–41. 10.6004/jnccn.2018.0020 PMC6476336 29632061

[13] Schmid-Bindert G, Vogel-Claussen J, Gutz S, Fink J, Hoffmann H, Eichhorn ME, et al. Incidental pulmonary nodules – what do we know in 2022. Respiration. 2022;101(11):1024–34. 10.1159/000526818 36228594 PMC9945197

[14] Eisenberg R, Bankier A, Boiselle P. Compliance with Fleischner Society guidelines for management of small lung nodules: a survey of 834 radiologists. Radiology. 2010;255(1):218–24. 10.1148/radiol.09091556 20308458

[15] Umscheid CA, Wilen J, Garin M, Goldstein JD, Cook TS, Liu Y, et al. National survey of hospitalists’ experiences with incidental pulmonary nodules. J Hosp Med. 2019;14(6):353–6. 10.12788/jhm.3115 30794135 PMC6824805

[16] Digby GC, Habert J, Sahota J, Zhu L, Manos D. Incidental pulmonary nodule management in Canada: exploring current state through a narrative literature review and expert interviews. J Thorac Dis. 2024;16(2):1537–51. 10.21037/jtd-23-1453 38505054 PMC10944736

[17] Rampinelli C, Cicchetti G, Cortese G, Polverosi R, Farchione A, Iezzi R, et al. Management of incidental pulmonary nodule in CT: a survey by the Italian College of Chest Radiology. Radiol Med (Torino). 2019;124(7):602–12. 10.1007/s11547-019-01011-1 30859388

[18] McWilliams A. Probability of cancer in pulmonary nodules detected on first screening CT. New Engl J Med. 2013;369(10):910–19. 10.1056/NEJMoa1214726 24004118 PMC3951177

[19] Herder GJ, van Tinteren H, Golding RP, Kostense PJ, Comans EF, Smit EF, et al. Clinical prediction model to characterize pulmonary nodules: validation and added value of 18F-fluorodeoxyglucose positron emission tomography. Chest. 2005;128(4):2490–6. 10.1378/chest.128.4.2490 16236914

