# Supplementary material for: Adherence to guidelines for incidental pulmonary nodules: insights from a Nordic survey
Source: Acta Oncol. 2025 Jan 8;64:42461. doi: 10.2340/1651-226X.2025.42461 (PMC11734304; doi:10.2340/1651-226X.2025.42461)
Supplement: Adherence to guidelines for incidental pulmonary nodules: insights from a Nordic survey [file AO-64-42461-s1.pdf]

Supplementary material has been published as submitted. It has not been copyedited, or typeset by Acta Oncologica

Supplementary

Nordic survey on pulmonary nodule management

---

1) **Which country are you based in?**

Denmark; Finland; Norway; Sweden

---

2) **Which city are you based in?**

Name of city:_________________________

---

3) **Does your country have national guidelines for managing lung nodules?**

Yes / No

---

4) **What type of guidelines do you primarily follow for managing lung nodules?**

National guidelines / International guidelines (please specify) / Local or institutional guidelines/ Other (please specify)

---

5) **How many new lung nodules does your institution typically start to follow per month?** (Open answer)

---

6) **How many lung nodules does your institution currently follow (approximately)?**

Open answer

---

7) **How many lung cancers are diagnosed at your institution annually?**

Open answer

---

8) **Which departments are managing lung nodules at your institution**

Respiratory Medicine Department exclusively; Radiology Department exclusively; Mostly Respiratory/Radiology department, but other departments also manage them (Department that detects lung nodule/GP etc.)

---

9) **How would you manage a solid lung nodule of 6 mm with no previous imaging and no typical benign morphology?**

Investigation with PET and biopsy; CT follow-up 3 months; CT follow-up 6 months; CT follow-up 12 months; Depending on Brock risk score

---

10) **How would you manage a solid lung nodule of 8 mm with no previous imaging and no typical benign morphology?**

Investigation with PET and biopsy; CT follow-up 3 months; CT follow-up 6 months; CT follow-up 12 months; Depending on Brock risk score

---

11) **How would you manage a ground glass opacity (GGO) of 8 mm with no previous imaging?**

Investigation with PET and biopsy; CT follow-up 3 months; CT follow-up 6 months; CT follow-up 12 months

---

12) **How would you manage a solid lung nodule of 10 mm with no previous imaging and no typical benign morphology?**

Investigation with PET and biopsy; CT follow-up 3 months; CT follow-up 6 months; CT follow-up 12 months; Depending on Brock risk score

---

13) **How would you manage a ground glass opacity (GGO) of 10 mm with no previous imaging?**

Investigation with PET and biopsy; CT follow-up 3 months; CT follow-up 6 months; CT follow-up 12 months
